# Novel Methods to Generate Active Ingredients-Enriched Ashwagandha Leaves and Extracts

**DOI:** 10.1371/journal.pone.0166945

**Published:** 2016-12-09

**Authors:** Sunil C. Kaul, Yoshiyuki Ishida, Kazuya Tamura, Teruo Wada, Tomoko Iitsuka, Sukant Garg, Mijung Kim, Ran Gao, Shoichi Nakai, Youji Okamoto, Keiji Terao, Renu Wadhwa

**Affiliations:** 1 Drug Discovery and Assets Innovation Laboratory, DBT-AIST International Laboratory for Advanced Biomedicine (DAILAB), National Institute of Advanced Industrial Science & Technology (AIST), Tsukuba, Japan; 2 CycloChem Co., Ltd., 7-4-5 Minatojima-minamimachi, Chuo-ku, Kobe, Japan; 3 DAI-DAN Co., Ltd., 390 Kitanagai, Miyoshi-machi, Iruma-gun, Saitama, Japan; 4 Osaka Prefecture University, 1-1 Nakakugakuencho, Sakai-city, Osaka, Japan; 5 School of Integrative and Global Majors, University of Tsukuba, 1-1-1 Tennodai, Tsukuba, Japan; 6 Zuiron Private Ltd., 2-3-1 Nakajyosanjimacho, Tokushima-city, Tokushima, Japan; Indian Institute of Technology Delhi, INDIA

## Abstract

Ashwagandha (*Withania somnifera*) is an Ayurvedic herb commonly used in world-renowned traditional Indian home medicine system. Roots of Ashwagandha have been traditionally known to possess a variety of therapeutic and health promoting potentials that have not been sufficiently supported by laboratory studies. Nevertheless, most, if not all, of the preventive and therapeutic potentials have been assigned to its bioactive components, steroidal alkaloids and lactones. In contrast to the traditional use of roots, we have been exploring bioactivities in leaves of Ashwagandha. Here, we report that the leaves possess higher content of active Withanolides, Withaferin-A (Wi-A) and Withanone (Wi-N), as compared to the roots. We also established, for the first time, hydroponic cultivation of Ashwagandha and investigated the effect of various cultivation conditions on the content of Wi-A and Wi-N by chemical analysis and bioassays. We report that the Withanone/Withaferin A-rich leaves could be obtained by manipulating light condition during hydroponic cultivation. Furthermore, we recruited cyclodextrins to prepare extracts with desired ratio of Wi-N and Wi-A. Hydroponically grown Ashwagandha and its extracts with high ratio of withanolides are valuable for cancer treatment.

## Introduction

Plant extracts or their active ingredients constitute about ¼^th^ of the medicinal drugs. Herbal medicines have not only been known for their safety and efficacy, but also for their affordability and availability to the human populations that either do not have access to the modern medicine or can not afford them. According to World Health Organization (WHO), more than 80% of population in developing countries still relies primarily on traditional herbal remedies for a variety of diseases. *Withania somnifera* (Solanaceae family), commonly called Ashwagandha or Indian ginseng, is an important medicinal shrub that grows to about a meter in height on slightly basic and moist soil at optimal temperatures of 20–32°C. Extensively used in Indian, Unani and African traditional medicines, it has been categorized in GRAS (Generally Regarded As Safe) food substances by WHO [1–3]. Ashwagandha is well known for its enormous therapeutic potential for a large number of ailments, including stress, cardiac, brain and immune disorders, inflammation and cancer [2, 4–11]. Although mechanisms of these activities have not been clearly demonstrated by laboratory studies, several of these medicinal properties have been attributed to its wide array of secondary metabolites. These include alkaloids (tropine, psudotropine, 3-trigloyloxytropine, choline, anaferine, anahygrine and withanosomine), flavanol glycosides (6,8-dihydroxykaempferol 3-rutinoside, quercetin and 3-rutinoside-7-glucoside), glycowithanolides (sitoindoside VII to X steroidal lactones, withanolide A, withanolide D, withanone, withaferin A and withanone), sterols and phenolics [11–24].

In traditional home medicine, Ashwagandha roots have been commonly used for several kinds of herbal formulations, wherein predominant bioactives are Withaferin A, Withanolide A and Withanone [5,6,8,21]. We initiated to explore bioactivities in Ashwagandha leaves for the reasons, such as (i) to obtain ample supply without sacrificing the plants, (ii) to rule out soil contaminants, (iii) easy distinction of the healthy versus diseased plants, (iv) ease of cleaning and extraction processes and (v) avoid strong unpleasant odor of roots. We initially demonstrated that both alcoholic (i-Extract) and water extracts (WEX) of Ashwagandha leaves possess considerable anticancer activities. Active constituents for these bioactivities were identified as two main Withanolides, Withanone and Withaferin A in i-Extract, and triethylene glycol in WEX [25–28]. Mechanisms of action of such activities were determined by multiple approaches including loss-of-function screening, cDNA array, bioinformatics and molecular analyses. The data revealed that the two kinds of extracts possess different bioactive constituents and work through independent pathways involved in (i) activation of tumor suppressor genes, (ii) induction of oxidative stress and (iii) induction of DNA damage signaling [4,26–28]. Furthermore, *in vitro* anticancer activity of the alcoholic and water extracts was well translated to *in vivo* anti-tumor assays in nude mice wherein the tumor progression and metastasis were significantly suppressed. Based on these studies, we also formulated a combination of Withanone and Withaferin A with potent anti-metastasis activity [14]. Interestingly, we discovered that the low doses of leaf extracts protect normal cells against oxidative stress [29]. Similarly, biochemical and imaging assays in various *in vitro* neuronal cell oxidative stress models revealed that the extracts and the purified components (Withanone, Withanolide A from i-Extract, and triethylene glycol from WEX), when used at low dose, protected the glial and neuronal cells from oxidative stress [30–34]. They also caused differentiation of neuroblastoma cells to neurons *per se* [29,33,35]. Furthermore, combination of the extracts and active components were highly potent, endorsing the therapeutic merit of the combinational approach [29].

In view of these findings, we initiated to develop technologies to obtain Active Ingredients-Enriched (AIE, called i) Ashwagandha by manipulating its environmental conditions. We demonstrate, for the first time, that the (**a**) field raised Ashwagandha leaves possess high proportion of active Withanolides as compared to the roots, (**b**) hydroponic cultivation of Ashwagandha, and conditions for growing i-Ashwagandha with high content of active Withanolides and (**c**) new extraction method for high yield of Withanolides and with desired Wi-N and Wi-A ratio.

## Materials and Methods

### Ethics statement

All *in vivo* experiments were performed in accordance with the regulations and approval (Experimental Plan Approval #2013–025) of Animal Experiment Committee, Safety and Environment Management Division of National Institute of Advanced Industrial Science & Technology (AIST), Japan.

### Preparation of crude alcoholic extract of Ashwagandha leaves

Crude alcoholic extracts of roots and leaves were prepared for chemical analysis. Briefly, dried roots or leaf powder was suspended in 85% ethanol in a ratio of 1:30 and incubated at 85°C for 2 h in a reflux system. The collected extract was filtered and concentrated by evaporation at 60°C. The filtrate was lyophilized, by freeze-drying, for overnight. HPLC analysis of the extract was performed using Shimadzu HPLC system (LC-2010A) using YMC-Pack ODS-A (250 × 4.6 mm, 5 μm) column. Purified and well characterized Withaferin A and Withanone were used as standards.

### *In vitro* cytotoxicity assay

Human normal fibroblasts (TIG-3) were procured from Health Science Research Resources Bank, Japan. Osteosarcoma (U2OS) and Fibrosarcoma (HT1080) cell lines were obtained from DS Pharma, Japan, and cultured in Dulbecco’s Modified Eagle’s Medium DMEM (Invitrogen)-supplemented with 10% fetal bovine serum in a humidified incubator (37°C and 5% CO_2_). Cells grown at 40–60% confluency were treated with different kinds of extracts as indicated. Cells were incubated at 37°C for 48 h following which cytotoxicity assay was estimated by MTT {3-(4,5-dimethylthiazol-2-yl)-2, 5-diphenyltetrazolium bromide} assay (Life Technologies) as described earlier [26]. For long-term cell viability, colony-forming assays were performed. One thousand cells were plated in 6-well plate, and cultured either in control or extract-supplemented medium. Cells were cultured for 8–10 days (when colonies appeared in control cultures) with regular change in medium on every alternate day.

### Hydroponic cultivation of Ashwagandha

Hydroponic cultivation was established in plant factory that consisted of culture chambers made of (i) heat-insulated panels for temperature-control, (ii) high precision air conditioner and air duct system for airflow and (iii) automated ultrasonic humidifier for moisture control (S1A Fig). The set-up was designed to supply temperature-controlled air into the culture chambers. CO_2_ concentration was monitored in the chambers with the help of sensors and was regulated by supplying liquid CO_2_. Culture medium (liquid) for plants, stored in the container on the lowest rack in the culture chamber, was supplied from watering system (circulating pump) that adjusted the ingredients in an automated manner. Alternatively, pretreated seeds were sown in rockwool granule. Four weeks after seeding, the transplants were transferred to hydroponic system under 27/22°C or 25°C (as indicated) in light/dark period with 16 h light period and grown for 6 weeks. Stress treatments were started one-two weeks (as indicated) before harvest (Table 1).

**Table 1:** Hydroponic culture conditions.

**Normal**				
**Experimental condition**	**Default**	**Remarks**		
Fertilizer	1U	A-type from OAT Agrio Co., Ltd. (EC dS/m—2.4 and pH—6.15)		
Temperature (light/dark)	25/25°C			
Light source	HEFL lamp	HEFL lamp from Nippon Advanced Agri Co., Ltd.		
Light intensity	256 u mol/m^2^s	Distance from light source ~ 5 cm		
Light/Dark cycle	16/8 h			
Relative humidity	Not controlled			
**Stress**				
Fertilizer	Control	Enshi standard culture fluid (EC-2.4dS/m; N-16, P-4, K-8, Ca-8 and Mg-4 me/L)	EC dS/m	pH
			2.47	6.37
	Modified	Modified IV standard culture fluid	2.68	6.4
	2U	2U culture fluid	4.62	5.97
	4U	4U culture fluid	8.52	5.58
	NaCl_2U	Standard nutrient solution + NaCl 1,400 ppm	5.08	6.34
	NaCl_4U	Standard nutrient solution + NaCl 4,200 ppm	9.98	6.29
	CaCl_2__4U	Standard nutrient solution + CaCl_2_ 4,000 ppm	9.42	6.06
	MgSO_4__4U	Standard nutrient solution + MgSO_4_/7H_2_O 8,860 ppm	6.35	6.06
Temperature (light/dark)	27/22°C			
Light source	Fluorescent lamp			
Light intensity	150 u mol/m^2^s	Distance from light source ~ 42 cm		

#### Culture racks

Outline of culture racks is shown in (S1B Fig). Culture fluid was supplied to plants periodically by circulation pump (on/off regulated by a timer). Plants were settled in container (pot) filled with culture medium (rockwool granule procured from Nippon Rockwool Corporation, commonly used for strawberry).

#### Nutrient solution (irrigation system)

Adjustment of nutrient solution was done in the ingredient-mixing tank (100 L). Electric conductivity (EC) and pH were controlled to the default settings. Fertilizer A and Fertilizer B were used to make nutrient solution. Herein, liquid fertilizer by OAT-Agrio (A type: OAT-House #1 and #2) was used just as in most plant factories. Concentration of the nutrient solution was adjusted according to EC and the actual plant culture was done using 1 to 1/4 unit of standard concentration, depending on the growing speed (1 unit corresponds to ca 2.4 dS/m EC, while 1/2 unit, ca 1.2 dS/m EC). Nutrient solution adjusted was supplied to circulation tank (25 L) of each rack by supplying pump from the ingredient-mixing tank. The culture fluid supplied to culture beds (which consist of ten or more pots) was recycled in order not to perturb environment. Levels of the nutrient solutions in tanks was controlled by electrodes, and if the nutrient solution became less due to absorption by plants, nutrient solution was supplied automatically from the ingredient mixing tank.

#### Illumination

Hybrid Electrode Fluorescent Lamp (HEFL) illumination (Nippon Advanced Agri Co. Ltd.) was used to supply four different light wavelengths (i) fluorescent, (ii) Red, (iii) Red:Blue -1:1 and (iv) Red:Blue:Green-1:1:1 (S1C Fig). HEFL emits light with the same principle as fluorescent bulb, and uses exterior electrode tube that is used for large liquid crystal screen TV. Energy consumption is lower as compared to other illumination methods. Since heat generation is also low, close illumination to the plants was possible. It was also suitable for growing plants in a multiple layers and offered large cultivation area [36].

### Flow of culture experiments

Culture experiments were performed according to the flow shown in S1D Fig. Seeds were sown after pre-treatment that consisted of cleaning with water and treatment with gibberellin and low temperature. After about one week, when germination took place, seedlings were grown for about three weeks in culture fluid and transplanted on culture rack in culture chamber. Thus the hydroponic cultivation was started. Two weeks post-transplantation, nutrient solution (one unit of standard concentration) was supplied, and hydroponic culture was continued. The plants were harvested two months post-transplantation. Temperature of the culture chamber was adjusted to 25°C both for light (16 h) and dark (8 h) periods. CO_2_ concentration in the light period was controlled to ca. 1000 ppm (Table 1).

### Preparation of cyclodextrin-assisted water extract of Ashwagandha leaves

Water extract (10% w/v) was prepared from Ashwagandha dried leave powder, as described earlier, by overnight extraction in sterile water at 40°C with slow shaking [25]. For cyclodextrin (CD)-assisted aqueous extraction of Ashwagandha leaves (CD-WEX), the dried leaf powder (10% w/v) was mixed with aqueous solution of alpha (10%) or beta (2%) or gamma (10%) CD. The mixture was stirred for 24 h at 37°C with slow shaking (90 rpm) in TAITEC Bio-Shaker BR-43FL. The slurry was centrifuged at 3500 rpm for 10 min and the supernatant was filtered through 0.45-micron filter. The filtrate (CD extract) was subjected to HPLC and bioassays. High Withanone: Withaferin A ratio was identified in the residual precipitate of gamma CD extraction. In order to investigate anticancer potential of these extracts, the active components were extracted from precipitate in DMSO as described above. The supernatant (DM extract) obtained after centrifugation at 3500 rpm for 10 min was filtered through 0.45-micron filter and used for cytotoxicity assays. CD and DM extracts, obtained from dry leaf powder (10% by weight), were considered 100% and added to the cell culture medium in a range of 0.01~1% that corresponded to 10 μg ~1 mg/ml of leaf powder, respectively.

### High-pressure liquid chromatography (HPLC)

The HPLC for alpha, beta and gamma CD-assisted water extract of Ashwagandha (WEX) preparations and the gamma CD-complex was performed using Shimadzu HPLC system (LC-2010C). Phenomenex HPLC column (Luna 5 u C18 (2) 100 A: 4.60 mm I.D. x 150 mm) was used and the fractionation was performed at 45°C using Solution A: H_2_O (1% MeOH) and Solution B: methanol:ethanol:isopropanol in the ratio of 52.25: 45.30: 2.45 with gradient program as follows. A: 65% → 55% (30 min, flow rate: 1 mL/min; Injection volume: 10 μl). Detection was performed at 220 nm.

### *In vivo* tumor formation assays

Balb/c nude mice (4 weeks old, female) were bought from Nihon Clea (Japan). Animals used for experimentation received human care. All *in vivo* experiments were performed in accordance with the institutional regulations as approved by animal experiment ethical committee. Mice were housed under pathogen free conditions under a 12 h dark/light cycle and fed with standard chow *ad libitum*. For anti-tumor assays, HT1080 cells (6 x 10^6^ cells suspended in 0.2 ml of growth medium) were injected into the nude mice subcutaneously (two sites per mouse). Control group was treated with 2% carboxymethyl cellulose (CMC). WEX group was fed with 500 mg WEX/Kg body weight and CD-WEX group was fed with 500 mg WEX and 0.625 mg gamma CD/Kg body weight. The treatment started on the 8th day post-injection of cells, and was carried out 12 times on alternate days. Tumor formation was monitored for a month. Predefined human endpoints were established according to National Institute of Advanced Industrial Science & Technology (AIST), Japan Committee on the Ethics of Animal Experiments. Criteria set for need to euthanize was the tumor size, physical appearance including sickness, distress or immobility. Maximum tumor size allowed was 20 mm at the largest diameter. None of the animals, in the present study, met any criteria that required euthanization. The volume of subcutaneous tumors was calculated as V = L X W^2^/2, where L was length and W was width of the tumor, respectively. For metastasis assay, the recipient mice were sacrificed by cervical dislocation, lungs were fixed in 4% formaldehyde and the tumor colonies were counted 5 weeks after tail vein injection. This assay was performed using three mice for each group, and repeated twice.

## Results and Discussion

**Fig 1:**
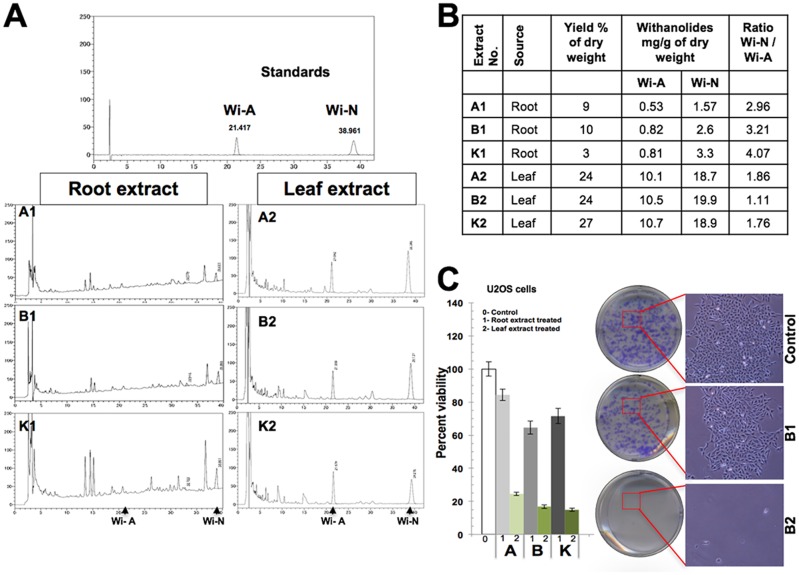
(**A**) HPLC analyses of alcoholic extracts of Ashwagandha roots (1) and leaves (2) raised at distant places, Ibaraki (K) and Tokushima (A and B) and Punjab, India (P). Purified Withaferin A (Wi-A) and Withanone (Wi-N) were used as standards. (**B**) Withanolide yield and ratio of Wi-N/Wi-A in extracts from root and leaves of Ashwagandha grown at distant places are shown. (**C**) Representative comparative cytotoxicity assay for root and leaf extracts of Ashwagandha. Cytotoxicity as determined by viability assay (short term cytotoxicity) and phase contrast images of cells in plates for colony forming assays (long term cytotoxicity) are shown.

Alcoholic extract of Ashwagandha leaves has been shown to possess anticancer activity in *in vitro* and *in vivo* assays [25–28,37]. Withanolide constituents present in the alcoholic extract of leaves, such as Withanone and Withaferin A, were shown to kill cancer cells by mechanisms involving apoptosis and growth arrest [25–28, 38–44]. Whereas Withanone causes selective cancer cell killing, Withaferin A, at high concentration, was seen to possess cytotoxicity to normal cells in *in vitro* assays. Addition of Withanone along with Withaferin A to the culture medium protected the normal cells against cytotoxicity of the latter [26]. Several NMR studies have shown that the Ashwagandha leaves collected from either different origins or different stages of development vary in the ratio of the Withanolides [45–48]. In order to explore the use of Ashwagadha for cancer treatment, we investigated the content of Wi-A and Wi-N in plants cultivated in Ibaraki and Tokushima (Japan) and Punjab (India). Comparative HPLC analyses of the alcoholic extracts of leaves and roots of these plants were carried out. The analyses revealed that the yield of Withanolides was several fold higher in leaves as compared to the roots raised at distant places (Fig 1A and 1B and S1E Fig). Cytotoxicity of these extracts for human cancer cells was investigated by short term and long term cell viability assays. Consistent with the high content of Withanolides in leaves, the leaf extracts showed higher toxicity to human cancer cells as compared to the root extracts (Fig 1C).

**Fig 2:**
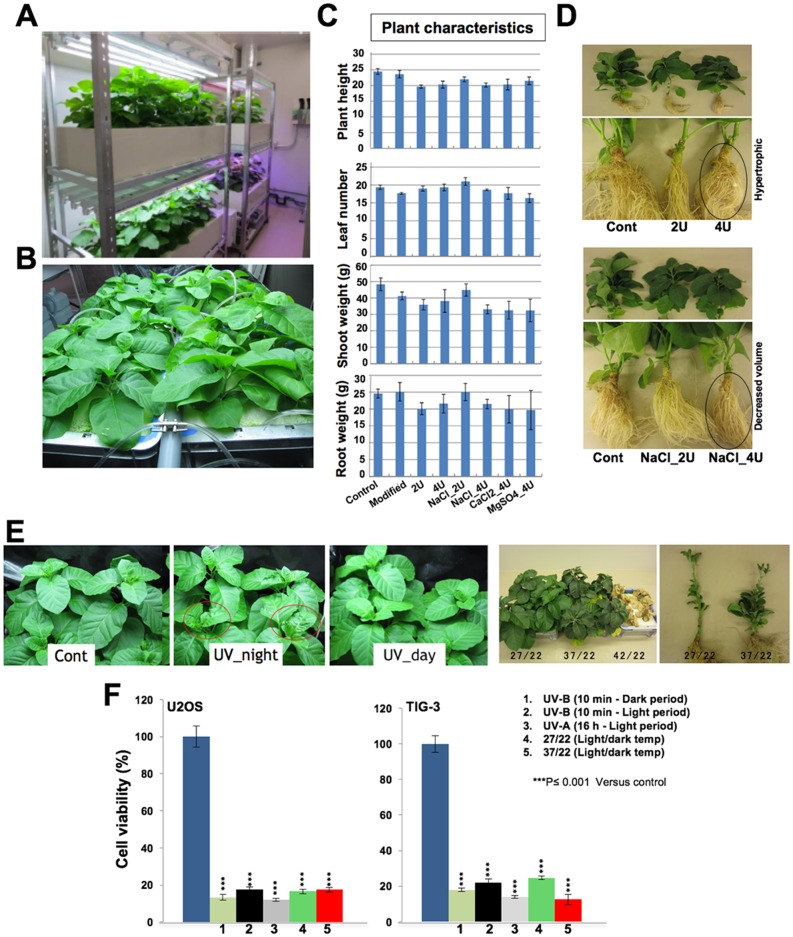
(**A**) Hydroponic cultivation system for Ashwagandha and (**B)** hydroponically grown leaves are shown. (**C**) Quantitative analysis of the effect of cultivation medium on plant characteristics showing no significance effect on the indicated attributes. (**D**) Cultivation in medium with 4 units caused hypertrophic roots, and addition of 4 units of NaCl caused decrease in root volume. (**E**) Effect of UV and temperature stress on hydroponically cultivated Ashwagandha. Exposure to UV-A during night caused leaf curling. Cultivation temperature 37/22 caused thick and dark green leaves, and 42/22 (light/dark) was lethal. (**F**) Cytotoxic assays of extracts (10 μg/ml) prepared from dried hydroponic leaves (100 mg/ml) cultivated under UV and temperature stress did not show any significant difference.

**Fig 3:**
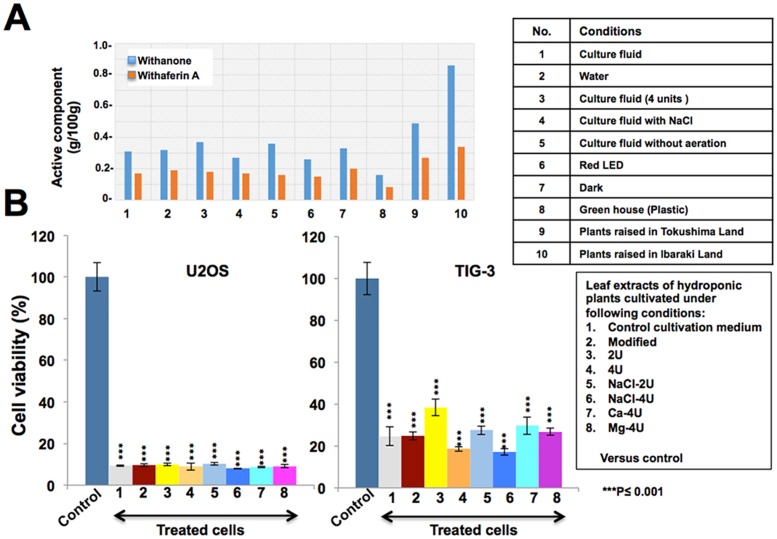
**(A)** Representative HPLC analyses of bioactives (Withanone and Withaferin A) in hydroponically grown Ashwagandha under different media and light conditions are shown. Leaves cultivated on land were used as controls. **(B)** Cytotoxicity assays of extracts (10 μg/ml) prepared from hydroponically grown Ashwagandha leaves (100 mg/ml). High Withanone content corresponded to selective toxicity to cancer cells.

Variations in chemotypes of Ashwagandha have been an issue for its use and value as a medicinal herb [45,47]. We envisaged that the hydroponic cultivation might be useful to provide uniform resource of bioactive Ashwagandha. In the present study, we demonstrate, for the first time, hydroponically grown Ashwagandha and evaluation of its leaves for anticancer bioactives. As shown in Fig 2A and S1 Fig, hydroponic system was set up. Under the conditions described in Material and Methods Section and S1 Fig, we successfully obtained hydroponically grown Ashwagandha, as shown in Fig 2A and 2B, and S1 Video. Furthermore, we investigated the effect of a variety of environmental conditions including, exposure to UV, temperature, pH and nutrients (Table 1). Plants grown under different conditions were examined for various attributes including, plant height, number of leaves, weight of shoots and roots (Fig 2C and 2D). Such analyses revealed that Ashwagandha is tolerant to a variety of stress conditions and did not show any dramatic changes in several plant attributes (Fig 2C). Some conditions that caused noticeable changes in root and leave attributes included (i) cultivation in four units of nutrient solution caused hypertrophic roots (Fig 2D), (ii) cultivation in nutrient solution containing four units of NaCl caused decrease in root mass (Fig 2D). Hydroponic plants cultivated in stressed conditions including exposure to UV and high temperature showed some visible alteration in growth. UV-B exposure for over 30 min/day caused dramatic leaf damage and death of plants. Therefore, UV-B exposure was restricted to 10 min. We noticed that the plants exposed to UV-B during night caused curling of leaves (Fig 2E). UV-A (16 h, during light period) exposure was well tolerated by the plants. Biological activity analysis of the leaf extracts from UV-stressed plants showed high toxicity to cancer cells suggesting that in spite of the above described phenotypic changes observed in the stressed plants, there was no major impact on anticancer bioactives. Similarly, we investigated the effect of high temperature stress. Light/dark (42/22°C) was lethal, (37/22°C) yielded shorter plants with more lateral shoot branching that possessed thick and dense green leaves. Similar to the UV-stressed, these plants also showed no difference in their cytotoxic activity in the extracts as compared to the control plants (Fig 2F). Leaves of hydroponically grown plants, under a variety of environmental conditions, were also examined for the content of Withaferin A and Withanone (Fig 3A). We found that similar to the leaves from plants cultivated on land, hydroponically grown plant leaves possessed higher content of Withanone than Withaferin A under all environmental conditions. Human cancer cells cytotoxicity assays revealed that the hydroponically grown leaves also possess anticancer activity. Furthermore, higher toxicity to cancer than to the normal cells was consistent with high Withanone content in the hydroponically grown leaves (Fig 3B).

**Fig 4:**
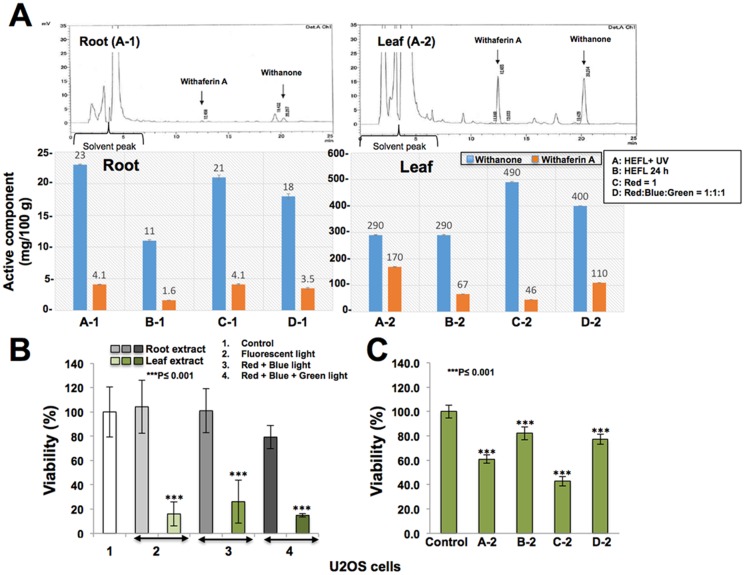
**(A)** HPLC analysis of roots (A-1) and leaves (A-2) from Ashwagandha plants cultivated under different light conditions. Quantitation of Withanone and Withaferin A in root and leaves of Ashwagandha cultivated under different light conditions. **(B)** Cytotoxicity of Ashwagandha root and leaf extracts (derived from 1 mg/ml of root or leaf powder) to human cancer (U2OS) cells showing higher cytotoxicity of the latter. **(C)** Cytotoxicity of leaf extracts (derived from 0.5 mg/ml leaf powder) from plants raised under different light conditions to human osteosarcoma.

We next investigated the effect of different light conditions on hydroponically grown plants (S1C Fig). As shown in Fig 4A, plants cultivated in different light conditions showed variable content of Withanone and Withaferin-A. Leaves in all cases possessed higher content of Withanone (12~22 fold) and Withaferin A (10–40 fold) as compared to the roots (Fig 4A). Furthermore, roots of the plants cultivated under continuous exposure to HEFL, with no dark cycle, showed lowest content of Withaferin A and Withanone, leaves from these plants showed Withanone level similar to that of the ones cultivated under HEFL (16 h) + UV (8 h) (Fig 4A). The amount of Withaferin A was significantly higher in the leaves of plants grown under HEFL+UV. Of note, leaves, not the roots, of plants cultivated under red light showed highest content of Withanone (Withanone:Withaferin A/10:1). These data suggested that (i) Withanolide content of Ashwagandha leaves can be manipulated by their cultivation under different light conditions, (ii) plants cultivated under red light possess leaves with high level of Withanone and (iii) plants cultivated under supplemental UV light possess leaves with high level of Withaferin A. These data suggested that the mixture of red and blue light may yield plants with high content of Withanone as well as Withaferin A. Indeed we found that the plants cultivated under Red+Blue+Green light (1:1:1 ratio) possess high level of Withanone as well as Withaferin A (Fig 4A). Similar to the plants raised on land, the root extract of hydroponically raised plants possessed less withanolides than the leaves (Fig 4A), and showed low cytotoxicity to cancer cells (Fig 4B). Furthermore, we found that the leaf extract from plants raised under red light had 10-fold higher Withanone than Withaferin A, and were highly cytotoxic to cancer cells (Fig 4C).

**Fig 5:**
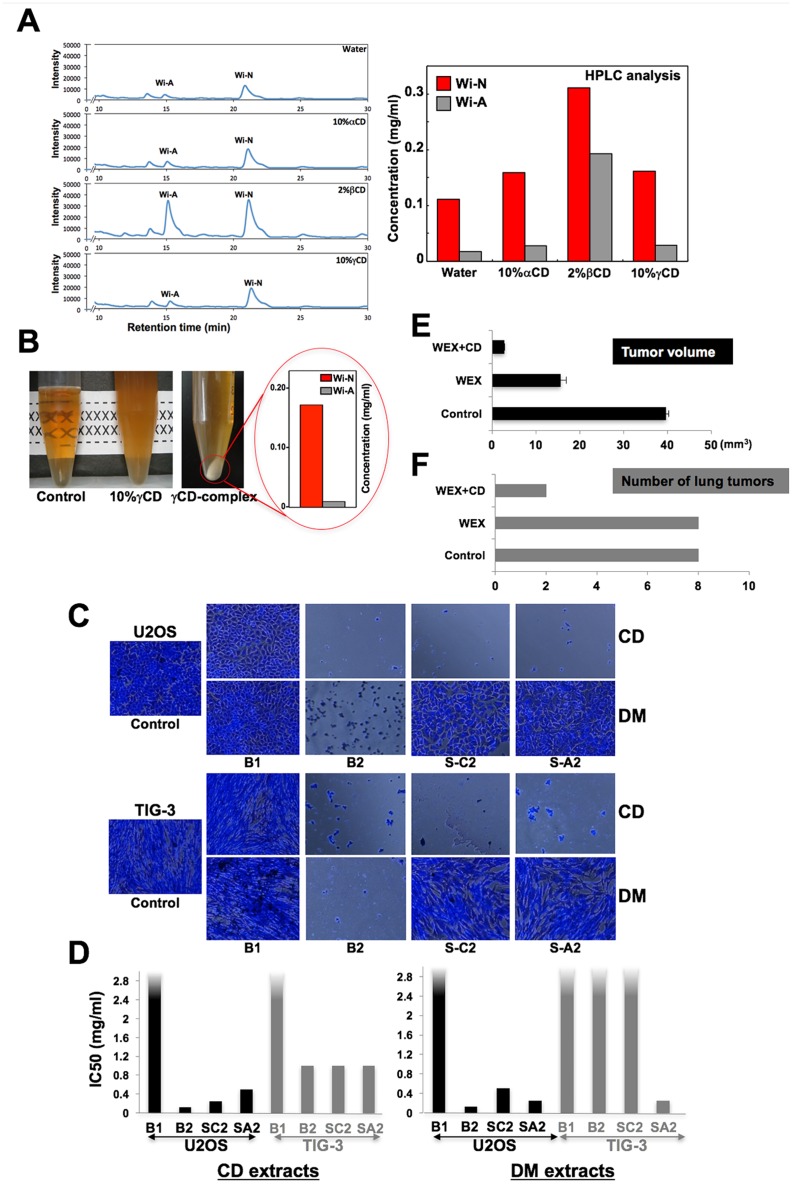
(**A**) Cyclodextrin-assisted water extractions of Ashwagandha leaves were performed and the level of Withanone and Withaferin A was determined by HPLC analysis. As shown, CD-assisted water extractions yield significant enrichment of Withanone and Withaferin A. (**B**) Residual gamma-CD precipitate analyzed by HPLC showed high ratio (17:1) of Withanone:Withaferin A. (**C**) *In vitro* cytotoxicity assay revealed that the CD extract of land raised Ashwagandha leaves (B2) was toxic to cancer as well as normal cells. CD-B1 (root) extract that possessed low content of Withanolides did not show activity. Gamma-CD residual precipitates (DM) that contained high level of Withanone showed higher cytotoxicity to cancer cells and were mild to normal cells. Leaves from hydroponically grown plants under the treatment of red (S-C2) and blue (S-A2) lights showed selective toxicity to cancer cells. (**D**) IC50 of each of the extracts obtained from several independent experiments is shown. DM extracts showed higher toxicity to the cancer than to the normal cells. (**E**) *In vivo* tumor formation assays in nude mice revealed that the gamma CD could enhance the anticancer potential in water extracts of Ashwagandha leaves.

In contrast to the alcoholic extract, water extract is more favorable due to the ease in preparation and compatibility with human food. Hence, the activities in the water extract were investigated [25]. We demonstrated that the water extract of Ashwagandha leaves possess anticancer activity. The active anticancer component was identified as triethylene glycol (TEG), in addition to low level of Withaferin A and Withanone, by chemical characterization including HPLC and NMR. These results predicted the need of novel extraction method(s) to obtain a mixture of alcohol- and water-soluble compounds in a moderate level for superior anticancer effects. In light of the above information, we investigated the potential of various isoforms of cyclodextrin (CD) for the preparation of aqueous anticancer extracts from Ashwagandha leaves. CDs are natural derivatives of starch or polymer of glucose that possess circular structure. These are widely used in food, pharmaceutical, agriculture, and environmental engineering and drug delivery because of its structure (hydrophobic inside and a hydrophilic outside) that enhances the solubility and bioavailability of compounds. Accordingly, aqueous extractions of hydrophobic drugs and health ingredients from plant materials by using CDs have been reported [49–51]. Gamma CD, widely accepted as food constituent, consists of 8 glucose monomers arranged in the form of a cyclic ring. It has been reported to enhance the bioavailability of hydrophobic ingredients such as coenzyme Q10 [52–54]. We recruited CDs for aqueous extraction of bioactives from Ashwagandha leaves. As shown in Fig 5A, we found that the CD-assisted aqueous extraction of Ashwagandha leaves resulted in an enrichment of anticancer Withanolides. Beta CD-derived extracts of Ashwagandha leaves contained highest level of Withanone and Withaferin A. By cell-based assays, we found that the CD extracts of Ashwagandha leaves have enhanced cancer cell cytotoxicity as compared to the conventional water extract. HPLC analysis of Withanone and Withaferin A in gamma-CD residual precipitate revealed that they contained 17-fold higher Withanone than Withaferin A (Fig 5B). We earlier reported that Withanone:Withaferin A (in the ratio of 20:1) possess high anticancer and anti-metastasis activities [14] and hence hypothesized that the gamma-CD residual precipitates could be very useful for cancer treatment. We validated by *in vitro* and *in vivo* experiments. For cell culture experiments, the bioactives in gamma-CD residual precipitate were extracted in DMSO (called DM extracts). Cell-based viability assays revealed that the CD extracts were more cytotoxic to both cancer and normal cells. Root extracts that contained low level of Withaferin A and Withanone were ineffective (Fig 5C). DM extracts showed higher cytotoxicity to cancer, and milder to normal, cells. Quantitative MTT assays endorsed that whereas root extracts (both CD and DM, with low level of Withanolides in each) had IC50 >2% for cancer and normal cells, the leaf-CD extracts were cytotoxic to both cancer (IC50 ~ 0.1%) and normal (IC50 ~ 1%) cells. Leaf-DM extracts showed higher toxicity to cancer cells (IC50 ~ 0.125% as compared to >2% for normal cells) (Fig 5D). The results were confirmed by extracts generated from hydroponically cultivated leaves exposed to (i) red light- high Withanone (S-C2) and (ii) UV- high Withaferin A (S-A2) (Fig 2). S-C2DM extracts from hydroponically cultivated plants showed selective toxicity to cancer cells. In *in vivo* nude mice tumor formation assays, aqueous extract of leaves and gamma CD combination caused stronger suppression of subcutaneous tumors and lung metastasis in nude mice (Fig 5E and 5F). Taken together, it is strongly suggestive that cyclodextrins are useful for aqueous extraction of bioactives in Ashwagandha leaves that could significantly enhance the anticancer activity *in vivo*.

## Conclusion

We have succeeded in establishing a hydroponic cultivation of Ashwagandha with enriched bioactives. We demonstrate that the leaves of Ashwagandha possess high content of bioactives and it could be further manipulated by light conditions during their cultivation. Whereas red light yielded leaves with high content of Withanone, UV light resulted in high level of Withaferin A. Furthermore, we have developed a new method of extraction for preparing Withanone-rich extracts that could be used in effective cancer treatment.

## Supporting Information

S1 Fig Schematic diagram of (**A**) the plant factory for hydroponic cultivation of Ashwagandha, (**B**) cultivation racks, pots and medium circulating system, and (**C**) Hybrid Electrode Fluorescent Lamp (HEFL) illumination system. Details of the set up are described in Materials and Methods Section. **(D)** Schematic flow of hydroponic cultivation of Ashwagandha. (**E**) Withanolide yield and ratio of Wi-N/Wi-A in extracts from the leaves of Ashwagandha raised in Punjab (P2).
(TIF)

S1 Video Time lapse growth recording of hydroponic Ashwagandha. (M4V)
