# Supplementary figures and images for: Novel Methods to Generate Active Ingredients-Enriched Ashwagandha Leaves and Extracts
Source: PLoS One. 2016 Dec 9;11(12):e0166945. doi: 10.1371/journal.pone.0166945 (PMC5147857; doi:10.1371/journal.pone.0166945)

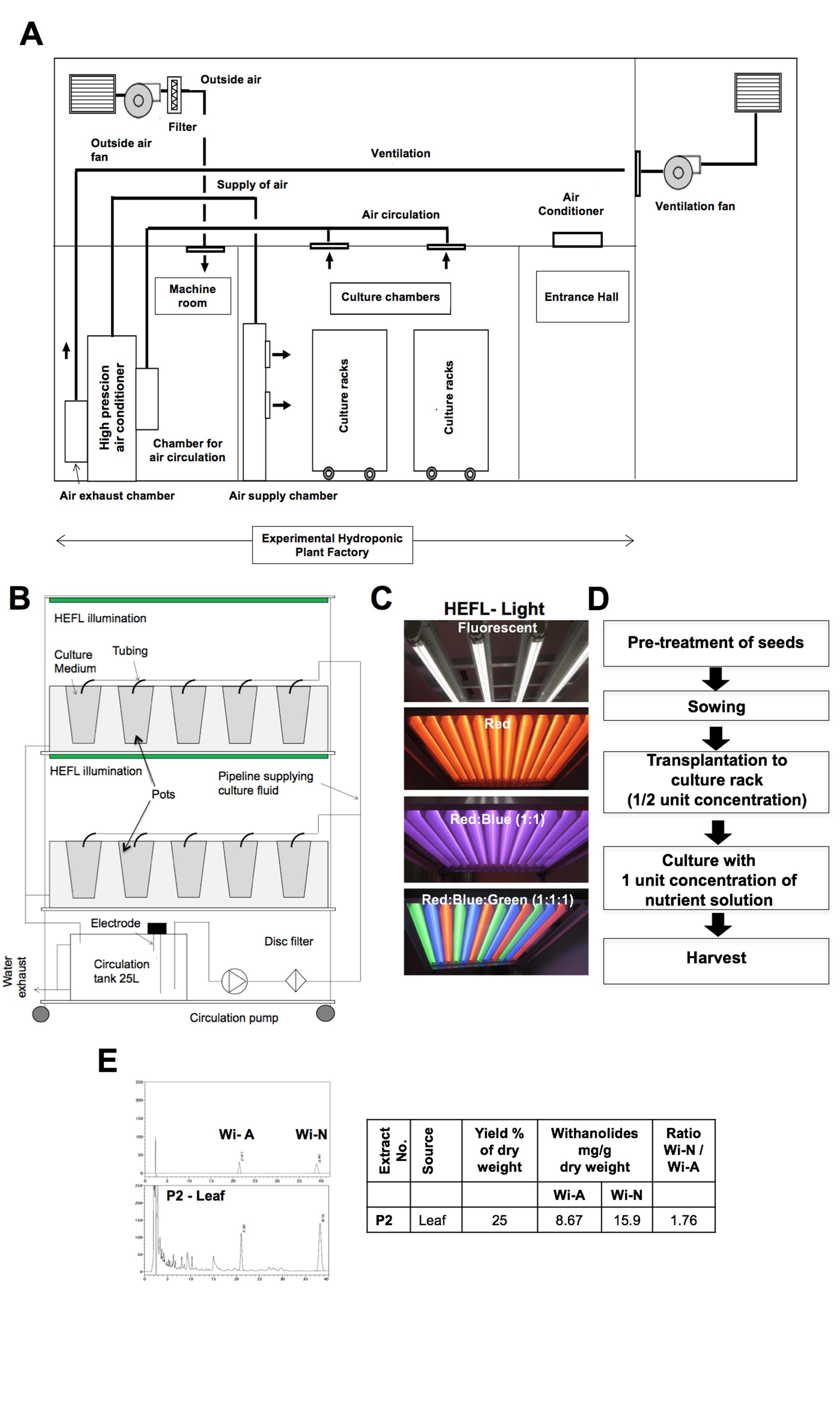

Supplement: S1 Fig — Schematic diagram of (A) the plant factory for hydroponic cultivation of Ashwagandha, (B) cultivation racks, pots and medium circulating system, and (C) Hybrid Electrode Fluorescent Lamp (HEFL) illumination system. Details of the set up are described in Materials and Methods Section. (D) Schematic flow of hydroponic cultivation of Ashwagandha. (E) Withanolide yield and ratio of Wi-N/Wi-A in extracts from the leaves of Ashwagandha raised in Punjab (P2). (TIF) [file pone.0166945.s001.tif]
